# Supplementary material for: Enhanced Antioxidant and Neuroprotective Properties of Pterostilbene (Resveratrol Derivative) in Amorphous Solid Dispersions
Source: Int J Mol Sci. 2024 Feb 28;25(5):2774. doi: 10.3390/ijms25052774 (PMC10932125; doi:10.3390/ijms25052774)
Supplement: Supplementary file 1 [file ijms-25-02774-s001.zip › ijms-2845672-supplementary.pdf]

Supplementary Materials

Enhanced Antioxidant and Neuroprotective Properties of Pterostilbene (Resveratrol Derivative) in Amorphous Solid Dispersions

Natalia Rosiak [1], Ewa Tykarska [2] and Judyta Cielecka-Piontek [1,*]

1 Department of Pharmacognosy and Biomaterials, Faculty of Pharmacy, Poznan University of Medical Sciences, 3 Rokietnicka St., 60-806 Poznan, Poland

2 Department of Chemical Technology of Drugs, Poznan University of Medical Sciences, 3 Rokietnicka St., 60-806 Poznan, Poland

* Correspondence: jpiontek@ump.edu.pl; Tel.: +48-61-641-8395

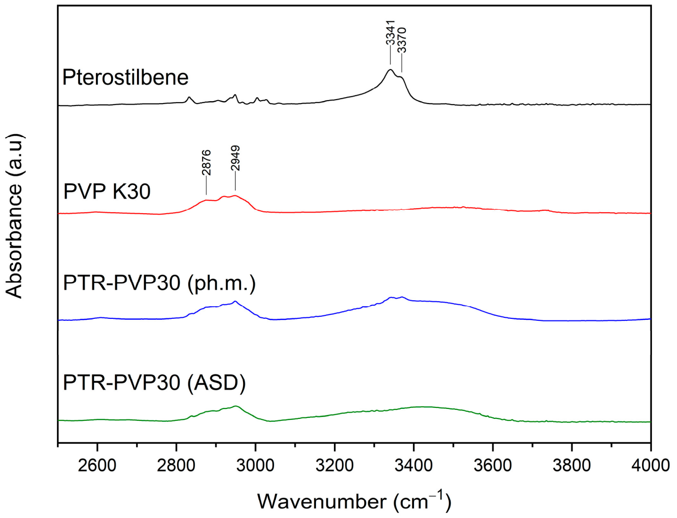

(**a**)

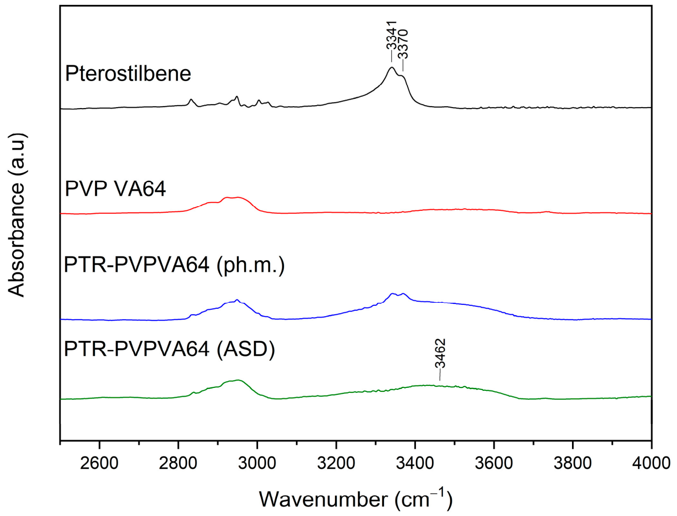

(**b**)

Figure S1. FTIR-ATR analysis, range 2550–4000 cm^{-1}: (**a**) pterostilbene (black line), PVP K30 (red line), pterostilbene-PVP30 physical mixture (blue line), pterostilbene-PVP30 amorphous solid dispersion (green line); (**b**) pterostilbene (black line), PVP VA64 (red line), pterostilbene-PVPVA64 physical mixture (blue line), pterostilbene-PVP VA64 amorphous solid dispersion (green line).

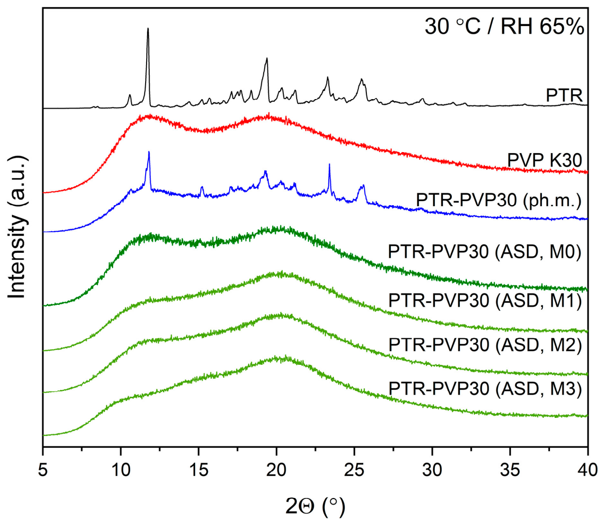

(**a**)

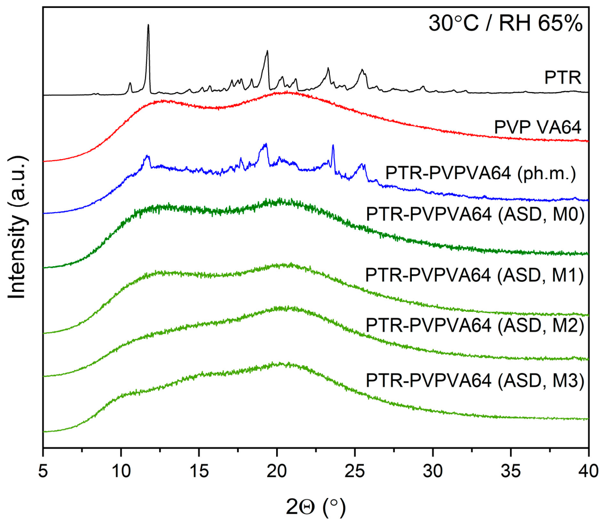

(**b**)

Figure S2. Physical stability studies (XRPD patterns, range 5–40° 2Θ): (**a**) pterostilbene (black line), PVP K30 (red line), pterostilbene-PVP30 physical mixture (blue line), pterostilbene-PVP30 amorphous solid dispersion (dark green line, M0), pterostilbene-PVP30 amorphous solid dispersion after 1-3 months (green line, M1–M3); (**b**) pterostilbene (black line), PVP VA64 (red line), pterostilbene-PVPVA64 physical mixture (blue line), pterostilbene-PVP VA64 amorphous solid dispersion (green line, M0), pterostilbene-PVPVA64 amorphous solid dispersion after 1–3 months (green line, M1-M3).

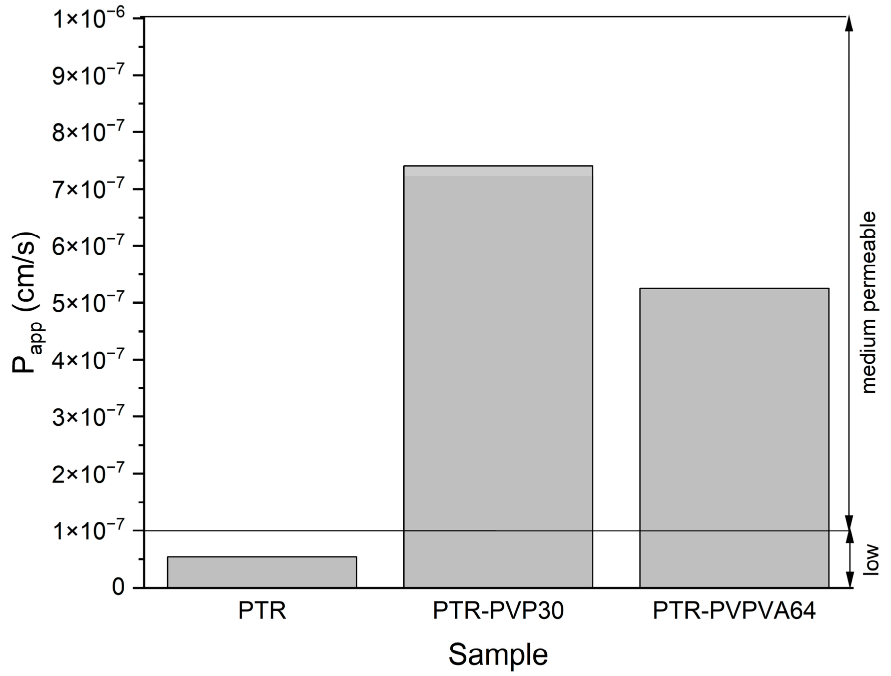

(**a**)

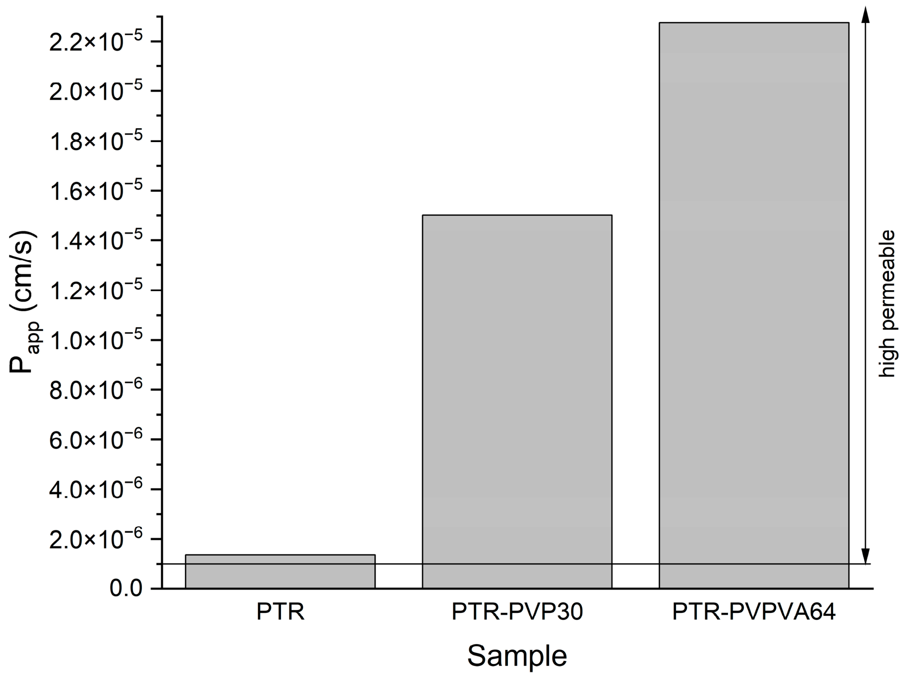

(**b**)

Figure S3. The results of PAMPA (**a**) GIT and (**b**) BBB assays. Legend: PTR – pterostilbene, PTR-PVP30 – amorphous solid dispersion of pterostilbene-PVP30, PTR-PVPVA64 – amorphous solid dispersion of pterostilbene-PVPVA64. Compounds designated as moderately permeable fall within the range of 0.1×10^{-6} cm·s^{-1} $\leqslant$ Papp < 1×10^{-6} cm·s^{-1}, while those with a Papp value $\geqslant$ 1×10^{-6} cm·s^{-1} are classified as highly permeable [1,2].

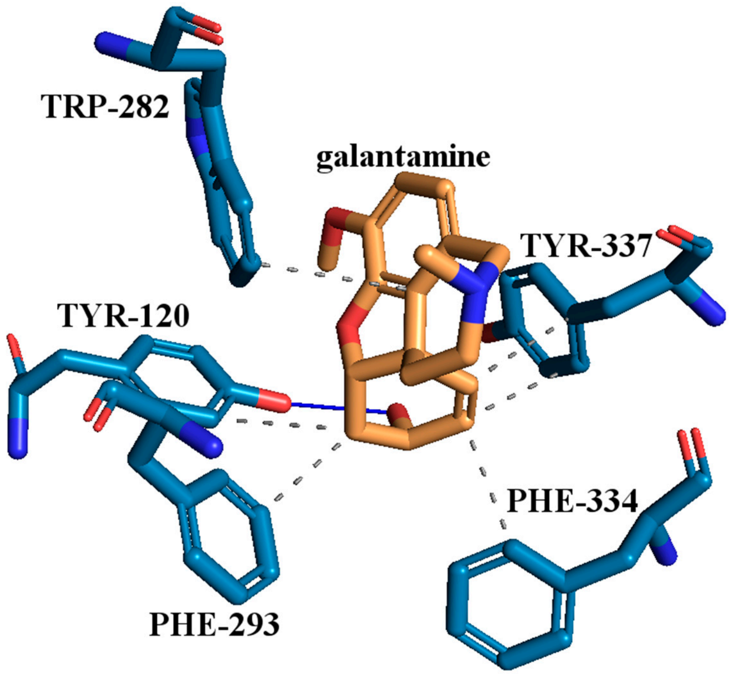

(**a**)

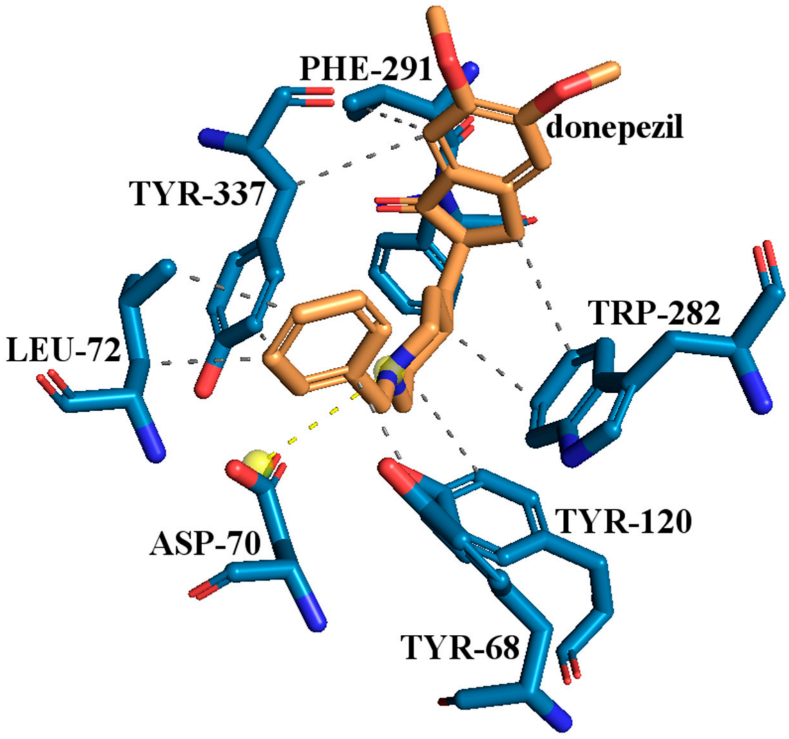

(**b**)

Figure S4. Proposed binding mode of positive control (**a**) galantamine (GAL), (**b**) donepezil (DON) with human acetylcholinesterase (AChE, PDB id: 4BDT). The key interactions of positive control with residues in the active sites of AChE. Legend: ASP - aspartic acid, LEU – leucine, PHE - phenylalanine, TRP - tryptophan, TYR - tyrosine, grey dashed line - hydrophobic interaction, blue solid line – hydrogen bond, yellow dashed line – salt-bridge.

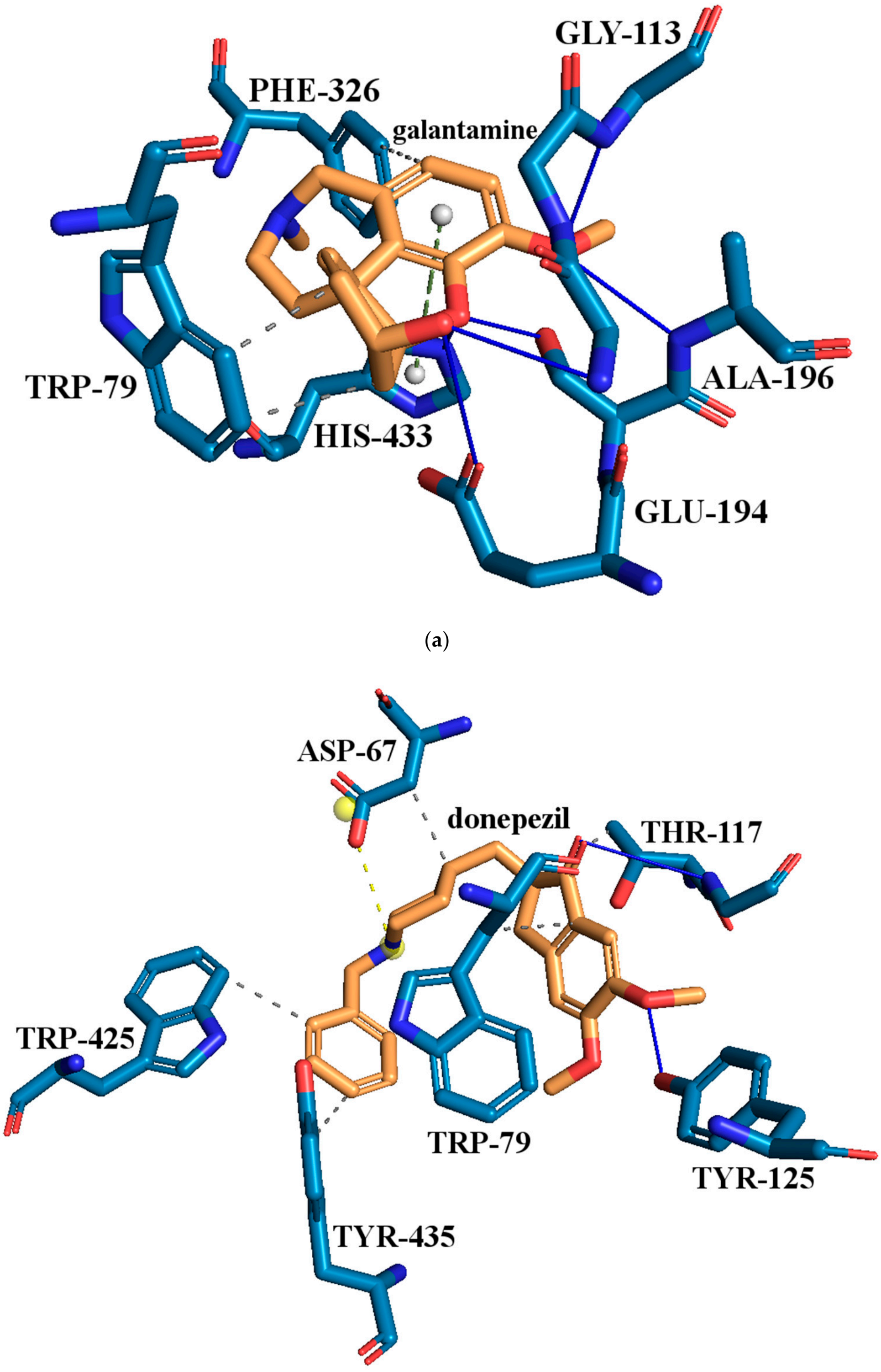

(**a**)

(**b**)

Figure S5. Proposed binding mode of positive control (**a**) galantamine (GAL), (**b**) donepezil (DON) with human butyrylcholinesterase (BChE, PDB id: 4BDS). The key interactions of positive control with residues in the active sites of BChE. Legend: ALA - alanine, ASP - aspartic acid, GLU - glutamic acid, GLY - glycine, HIS - histidine, PHE - phenylalanine, THR - threonine, TRP - tryptophan, TYR - tyrosine, grey dashed line - hydrophobic interaction, blue solid line – hydrogen bond, yellow dashed line – salt-bridge, green dashed line - π-stacking.

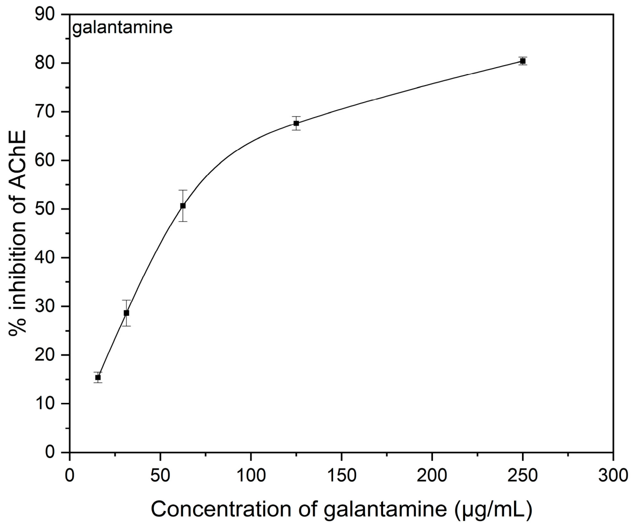

(**a**)

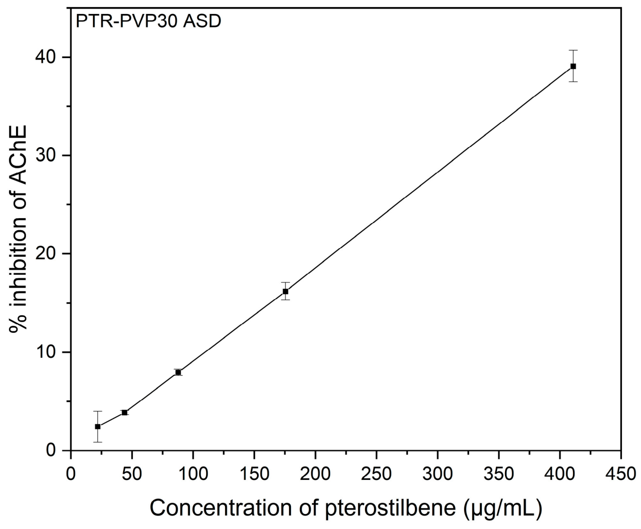

(**b**)

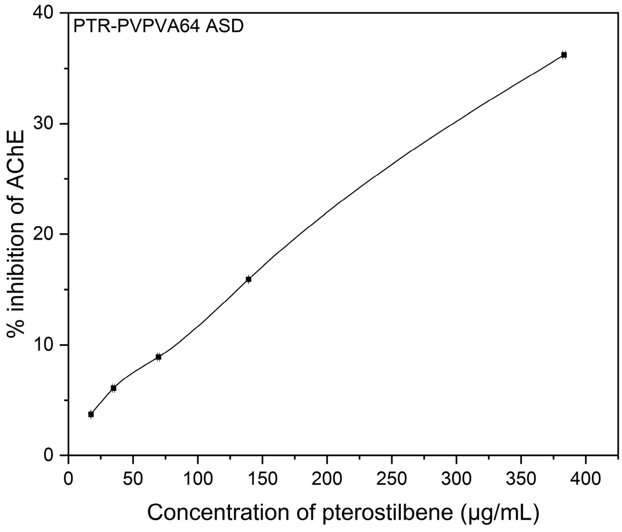

(**c**)

Figure S6. Acetylcholinesterase (AChE) inhibition dose-response curves of (**a**) galantamine, (**b**) pterostilbene in PTR-PVP30 amorphous solid dispersion (PTR-PVP30 ASD), and (**c**) pterostilbene in PTR-PVPVA64 amorphous solid dispersion (PTR-PVPVA64 ASD).

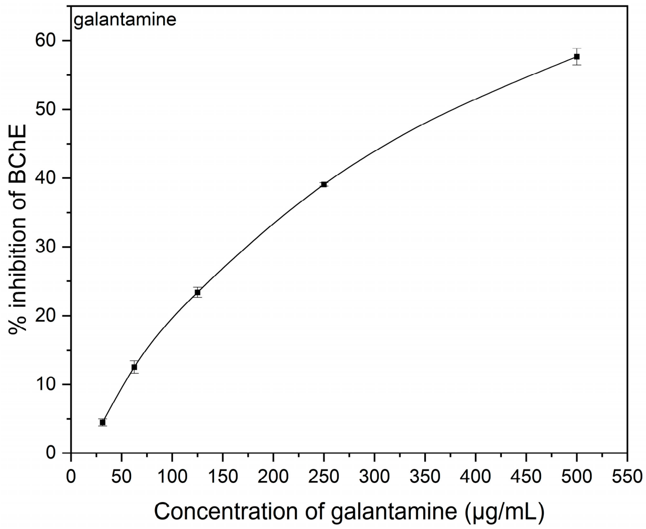

(**a**)

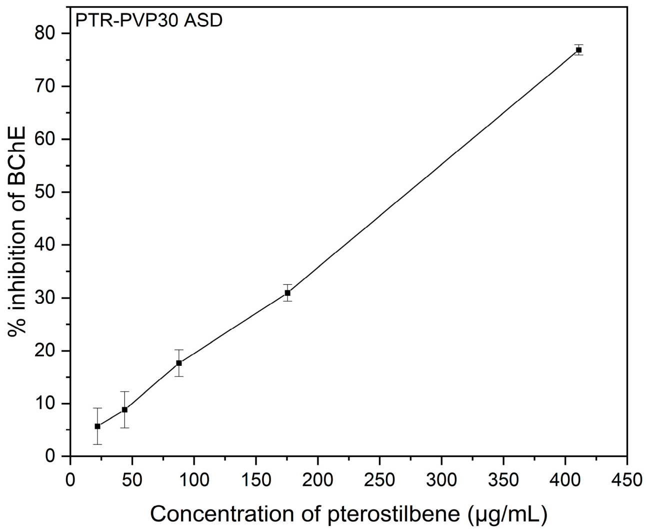

(**b**)

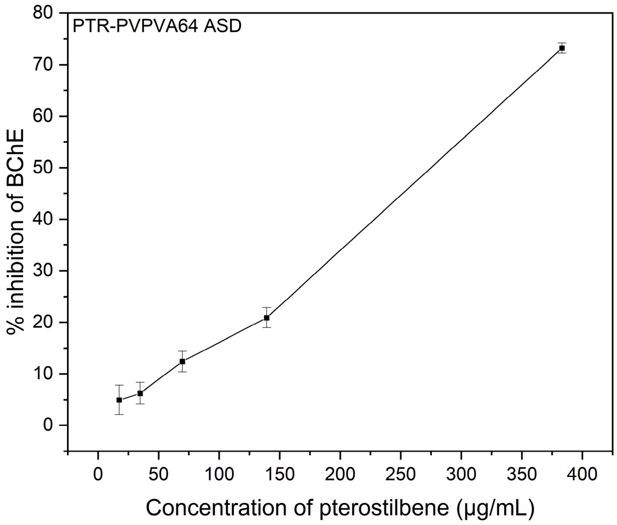

(**c**)

Figure S7. Butyrylcholinesterase (BChE) inhibition dose-response curves of (**a**) galantamine, (**b**) pterostilbene in PTR-PVP30 amorphous solid dispersion (PTR-PVP30 ASD), and (**c**) pterostilbene in PTR-PVPVA64 amorphous solid dispersion (PTR-PVPVA64 ASD).

Table S1. IC_{50} values for activities towards acetylcholinesterase (AChE) and butyrylcholinesterase (BChE) for pterostilbene (PTR), PTR-PVP30 amorphous solid dispersion (PTR-PVP30 ASD), PTR-PVPVA64 amorphous solid dispersion (PTR-PVPVA64 ASD), galantamine, and donepezil.

	**AChE**		**BChE**		**Selectivity for**	
	**IC_{50} (µg/mL)**	**IC_{50} (nM)**	**IC_{50} (µg/mL)**	**IC_{50} (nM)**	**AChE[a]**	**BChE[b]**
PTR	none	none	none	none		
PTR-PVP30 ASD	527.1±1.2	2056.6±4.6	268.0±3.5	1045.7±13.5	0.51	1.97
PTR-PVPVA64 ASD	540.8±3.8	2110.2±14.8	269.1±3.4	1050.0±13.2	0.50	2.01
galantamine	66.3±3.9	230.8±13.6	377.9±43.8	1315.2±152.4	5.70	0.18
donepezil*	2.5	6.7	2808.2	7400	1104.47	0.0009

* - donepezil IC_{50} values based on literature report [3]; a - selectivity for AChE was defined as IC_{50}(BChE)/IC_{50}(AChE); b - selectivity for BChE was defined as IC_{50}(AChE)/IC_{50}(BChE).

References

1. Fischer, H.; Kansy, M.; Avdeef, A.; Senner, F. Permeation of permanently positive charged molecules through artificial membranes—influence of physico-chemical properties. Eur. J. Pharm. Sci. 2007, 31, 32–42, doi:10.1016/j.ejps.2007.02.001.

2. Garbiec, E.; Rosiak, N.; Zalewski, P.; Tajber, L.; Cielecka-Piontek, J. Genistein Co-Amorphous Systems with Amino Acids: An Investigation into Enhanced Solubility and Biological Activity. Pharmaceutics 2023, 15, 2653, doi:10.3390/pharmaceutics15122653.

3. Sugimoto, H.; Ogura, H.; Arai, Y.; Iimura, Y.; Yamanishi, Y. Research and development of donepezil hydrochloride, a new type of acetylcholinesterase inhibitor. Jpn. J. Pharmacol. 2002, 89, 7–20.
